# Distribution and analysis of subacromial spurs and the relationship with acromial classification and angle in healthy individuals

**DOI:** 10.1371/journal.pone.0301066

**Published:** 2024-03-28

**Authors:** Weichong Dong, Kezheng Du, Bo Shi, Tianci Wang, Bo Lu, Zhiyong Hou, Yingze Zhang, Jialiang Guo

**Affiliations:** 1 The School of Medicine, Nankai University, Tianjin, 300071, P. R. China; 2 Department of Pharmacy, The Second Hospital of Hebei Medical University, Shijiazhuang, 050051, P. R. China; 3 Department of Orthopaedics, Hebei Medical University Third Hospital, Shijiazhuang, 050000, P. R. China; 4 Chinese Academy of Engineering, Beijing, P. R. China; 5 NHC Key Laboratory of Intelligent Orthopeadic Equipment (Hebei Medical University Third Hospital), Shijiazhuang, 050000, P. R. China; AIIMS: All India Institute of Medical Sciences, INDIA

## Abstract

**Background:**

Subacromial spurs are considered the one of the pathology underlying shoulder impingement syndrome. Furthermore, few studies have focused on the morphology of the subacromial spurs in normal Chinese people. This study aimed to study the spur distribution and to illustrate the morphology of spurs, which may help guide the extent of acromioplasty.

**Methods:**

A total of 93 normal individuals were enrolled, and both shoulders of all enrolled individuals were analyzed. The subjects were divided and classified into three different groups by ages: group I = 18–40 years, group II = 41–60 years, and group III ≥ 61 years. The osteophyte distribution, osteophyte area, subacromial surface area and osteophyte area/subacromial surface area ratio were measured and illustrated using Mimics and 3-matic software. The shape of the acromion was classified according to the Bigliani and Morrison classification system. The acromial angle was also classified. Then, the relationship between osteophytes, acromial classification and acromial angle was analyzed.

**Results:**

Type II (curved shape) was the most common type of acromion, and the hooked shape was a rare form. A significant increase in the left subacromial surface area in males was observed in group III compared with group I (*P* < 0.001) and group II (*P* = 0.004). The total spur/subacromial area ratio was significantly higher in group II than I. An obvious increase in the right subacromial area was observed in group III compared with group I (*P* = 0.004). Furthermore, there was a significant increase in the right spur area (*P* = 0.021) and total spur/subacromial area ratio (*P* = 0.006) in females in group II compared with group I. Fewer spurs were observed on the left than on the right side (p = 0.0482). One spur was most common among type II acromions (29/36) (80.56%) on the left side and the right side (34/52, 65.38%).

**Conclusions:**

Spurs osteophytes are mainly distributed with an irregular shape and mostly run through the medial and lateral sides of the subacromial surface in normal subjects. The characteristics of subacromial spurs are so diverse that a surgeon must conduct subacromial decompression completely based on the morphology of individual spurs.

## Background

Shoulder pain is one of the most common complaints at the hospital, third only to lower back and knee pain. It has been reported that shoulder impingement syndrome (SIS) accounts for 30–35% of shoulder disorders and is the leading cause of shoulder pain and disability [1–3]. SIS can be caused by different pathologies, such as a hooked or laterally downsloping acromion, subacromial osteophytic spurs, hypertrophy of the coracoclavicular ligament, and hypertrophic osteoarthritis of the acromioclavicular joint. Subacromial or acromioclavicular spurs can be observed in approximately 50% of SIS patients [4]. Mechanical impingement was also considered the main cause (95%) of rotator cuff (RC) tears by Neer [5]. Since then, acromioplasty, subacromial decompression, subacromial bursectomy and anterior-inferior acromioplasty have been proposed and traditionally performed for the treatment of SIS or RC tears ^(23)^. To date, various studies have shown excellent results for arthroscopic RC repair conducted together with arthroscopic subacromial decompression [1,3], and acromioplasty has remained the standard surgical approach for the management of impingement lesions.

Among the many causes of SIS, subacromial spurs are considered one of the main pathologies, and various studies have reported the relationship between SIS and subacromial spurs [6–8]. Neer [5,9] described the need for partial anterior acromioplasty by stating that impingement of the RC by subacromial spurs results in RC tears. Dai [10] reported that arthroscopic decompression in patients with subacromial impingement yielded better functional results than conservative treatment after ≥ 60 months of follow-up. However, a more comprehensive understanding of the pathogenesis led to the publication of several papers questioning the role of acromioplasty or even the presence of subacromial impingement [11–15]. Beard [11] concluded that the value of this operation for subacromial shoulder pain is not definite. Furthermore, a comparison of the long-term clinical outcomes in patients treated with RC repair and those who underwent RC repair along with subacromial decompression showed no additional benefits [16]. Kang [17] also reported that a delayed reduction in acromial thickness is a normal postoperative feature one year after arthroscopic acromioplasty, and that an exaggerated concave contour of the acromial undersurface is also observed in some patients, which is considered a new risk factor for RC tears. Lähdeoja [18] reported that spur size did not correlate significantly with shoulder function or pain, which was proposed based on findings that subacromial decompression had no benefit in terms of functional outcome or pain. Jäschke [6] found that shoulder function was not compromised by the presence of a subacromial spur in the absence of bursitis after studying 69 patients treated for SIS. Additionally, it was reported that rather than the type of acromion, other factors might cause impingement in the formation of RC pathology [19–21].

Therefore, conflicting results in these studies demonstrated that the spur distribution might not be related to an increased risk of SIS and RC tears. One of the reasons for this uncertainty is that adequate and reproducible radiographs are difficult to obtain, and accurate 3-dimensional data, such as measurements obtained by CT or directly, are more convincing. For example, the cassette tilt view was proposed for evaluating the size of a subacromial spur, but the result might be affected by the projection angle [22]. To determine the effect of spurs on SIS and conduct targeted subacromial decompression, a preoperative understanding of subacromial spurs or the true shape of the subacromial surface based on CT data is essential. Furthermore, relatively few studies have focused on the morphology of the subacromial surface and distribution of spurs in normal Chinese people. Therefore, this study aimed to study the morphology of spurs in normal people based on CT data and illustrate the true character and distribution of spurs, which may help guide the extent of decompression of the subacromial surface.

## Methods

Ninety-three consecutive normal subjects including 45 (48.4%) males and 48 (51.6%) females, with a mean age of 65.63 ± 9.22 years (range, 20–86 years), were enrolled in this retrospective clinical study between July 2022 and June 2023 at our hospital. Both shoulders of all enrolled individuals were included. Patients aged ≥ 18 years and ≤ 70 years were included, and written informed consent was obtained from all enrolled subjects. The study was submitted to and approved by the ethics committee of Hebei Medical University Third Hospital (2021-083-1). The subjects were divided and classified into three different groups based on age: group I = 18–40 years, group II = 41–60 years, and group III ≥ 61 years. The exclusion criteria were as follows: presence of a rheumatic disease, symptomatic osteoarthritis of the shoulder, and shoulder instability.

### Subacromial spur distribution construction and analysis

Healthy individuals, including 45 adult men (46.2±13.3 years) and 48 adult women (53.6 ±16.4 years) whose dominant hand was the right hand, underwent shoulder CT arthrography (Siemens, Germany, SOMATOM^®^ Emotion) at our hospital were enrolled, and their DICOM images were downloaded through the PACS. The data were then reconstructed with Mimics (21.0). In this study, the upper and lower thresholds for bone were set at 1600 (maximum) and 226 (minimum) Hounsfield units, respectively.

**Fig 1:**
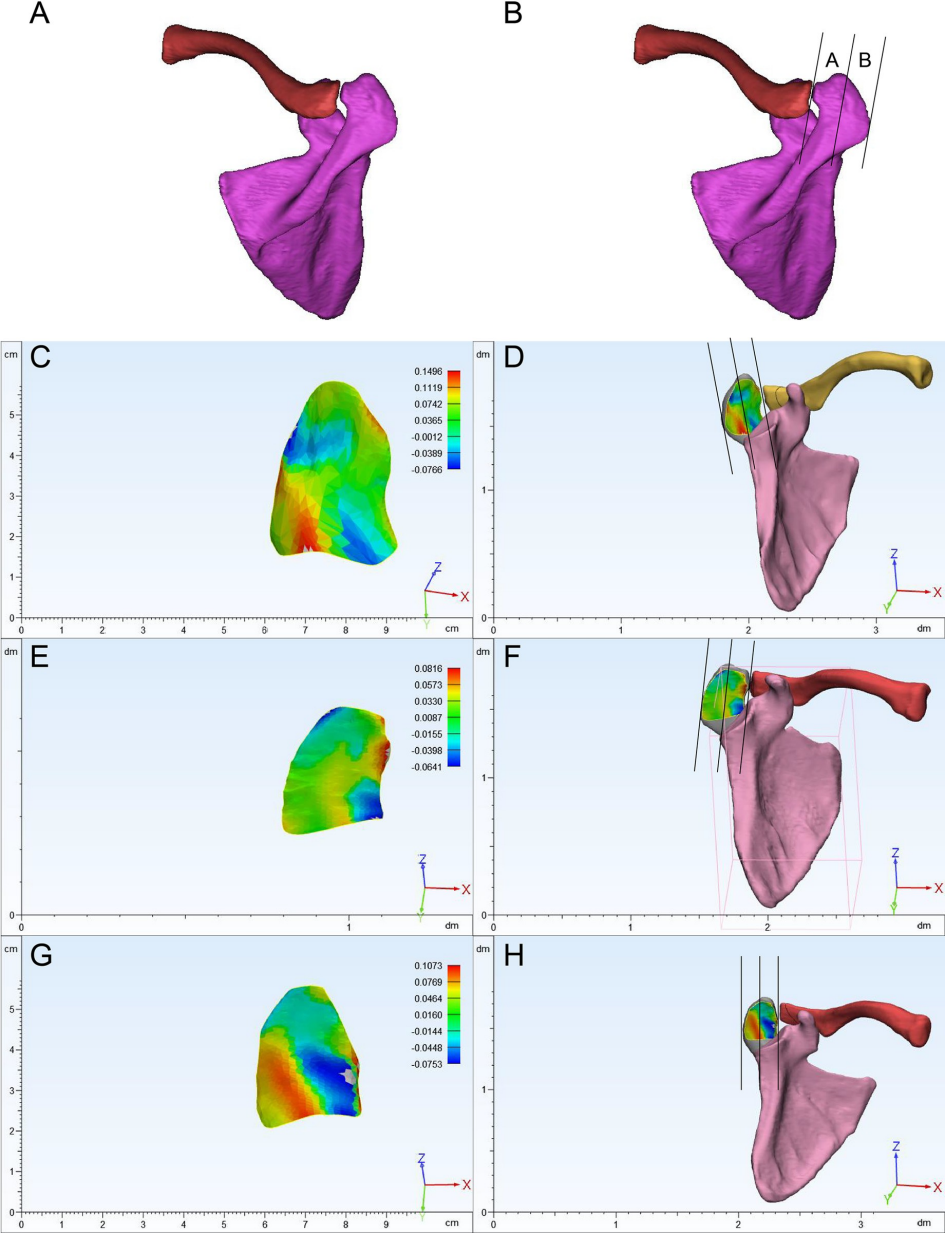
AB The acromion was divided into two equal parts illustrated on the upper surface. CD Spur located in the lateral region of the subacromial surface. EF Spurs located in the medial region of the subacromial surface. GH Spurs were located in the A+B region.

The 3D bilateral scapular bone images were reconstructed and exported directly into 3-matic (12.0). The region of the acromion was defined as the region located outside the line parallel to the acromioclavicular joint (S1 Video). The acromion was divided into two segments of equal width parallel to its longitudinal axis (regions A and B) (Fig 1).

**Fig 2:**
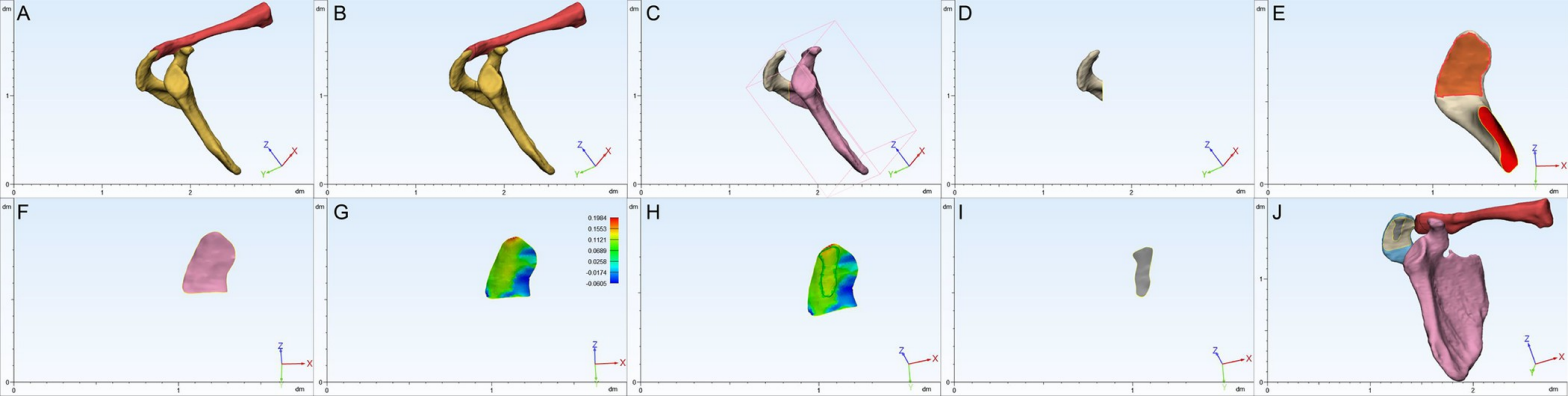
A 3D image obtained from Mimics. B The image was first divided using the rectangular patch tool in 3-matic software. C The clavicle was hidden, and the scapula was separated. D The body of the scapula was hidden, and the acromion was rotated. E The lasso area marking tool was used to demonstrate the subacromial region. F The smooth marking border function was then utilized to smooth the border of the segmented subacromial region. G Curvature analysis was conducted to assess the relative flatness of the inferior surface of the acromion (mesh type: Noisy, fitting radius: 8). H The lasso area marking tool and smooth marking border function were used again to obtain the spur border. I The area of the spur was obtained and calculated. J The last image obtained after a series of procedures in Mimics software.

**Fig 3:**
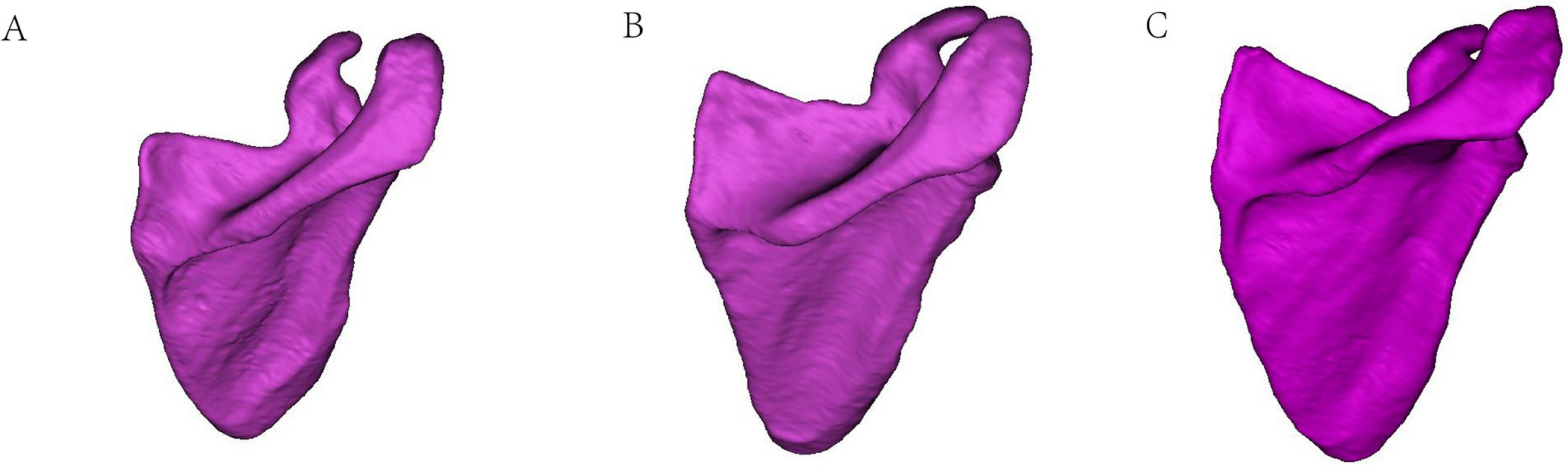
An L-shaped acromial angle: The lateral border of the acromion showed a bony protuberance, similar in shape to the letter L. B C-shaped acromial angle: The lateral border of the acromion had a curved arc shape. C Double angle-shaped acromial angle: The lateral border of the acromion showed two bony protuberances.

**Fig 4:**
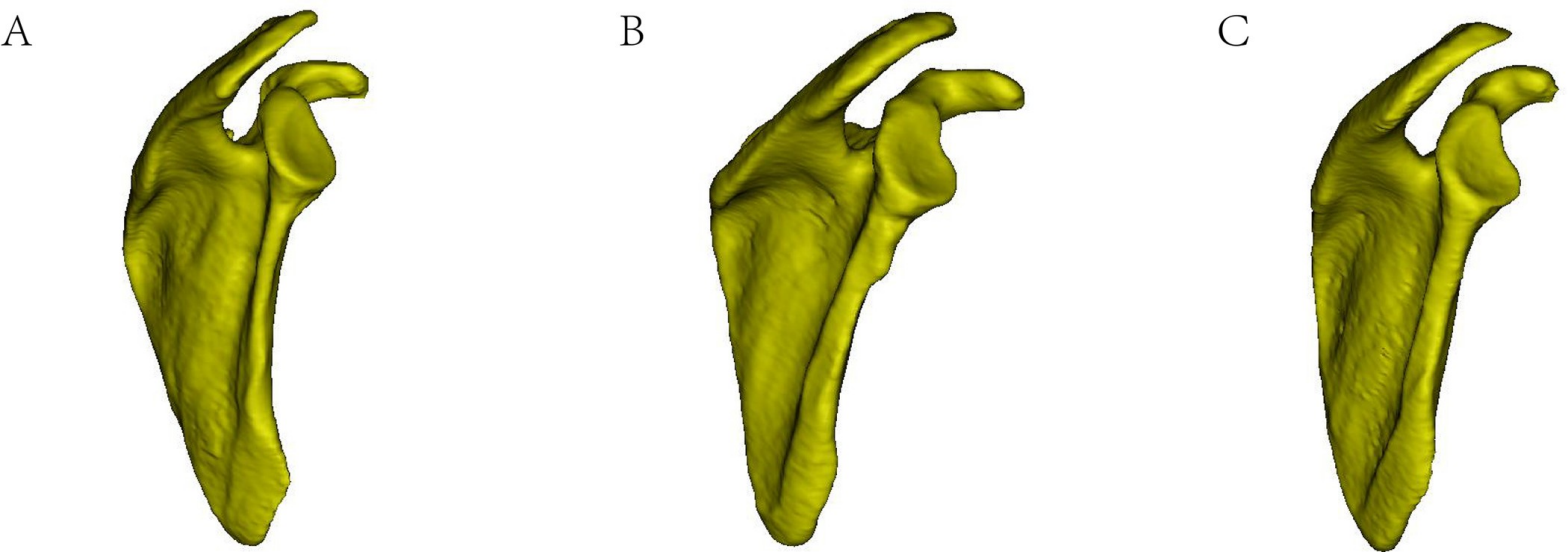
A Type I (flat) acromion. B Type II (curved) acromion. C Type III (hooked) acromion.

The osteophyte distribution, osteophyte area, subacromial surface area and the area ratio were measured and illustrated within 3-matic (12.0). The main tools used in the software were as follows: first, to divide and obtain the subacromial region of interest, the reconstructed 3D images in 3-matic software were divided with the rectangular patch tool (Fig 2A–2D). Second, the subacromial surface was identified with the lasso area and smooth border marking functions (Fig 2E and 2F). Third, curvature analysis was conducted to assess the flatness of the subacromial region (mesh type: noisy, fitting radius: 8) (Fig 2G). Fourth, the same function was used to obtain data regarding the morphological characteristics of subacromial spurs (Fig 2H–2J) (S2 Video). Furthermore, the acromial angle was classified as C-shaped, L-shaped, or double angle-shaped (Fig 3), and the acromion was classified as flat, hooked or curved according to the Bigliani [23] classification based on 3D images (Fig 4). The measurement results were calculated by two authors in our team, and the inter-observer reliability was calculated.

### Statistical analysis

Continuous data in this research are expressed as the mean ± standard deviation (SD). Two-tailed Student’s t test was used to evaluate differences between two independent groups. A p value ≤ 0.05 was considered to indicate a significant difference. All statistical analyses were performed with IBM SPSS Statistics 23.0 (SPSS, Chicago, Illinois, USA). The intraobserver reliability was statistically analyzed by weighted kappa coefficients (SPSS 21, IBM, Armonk, NY, USA).

## Results

### Acromial classification

**Fig 5:**
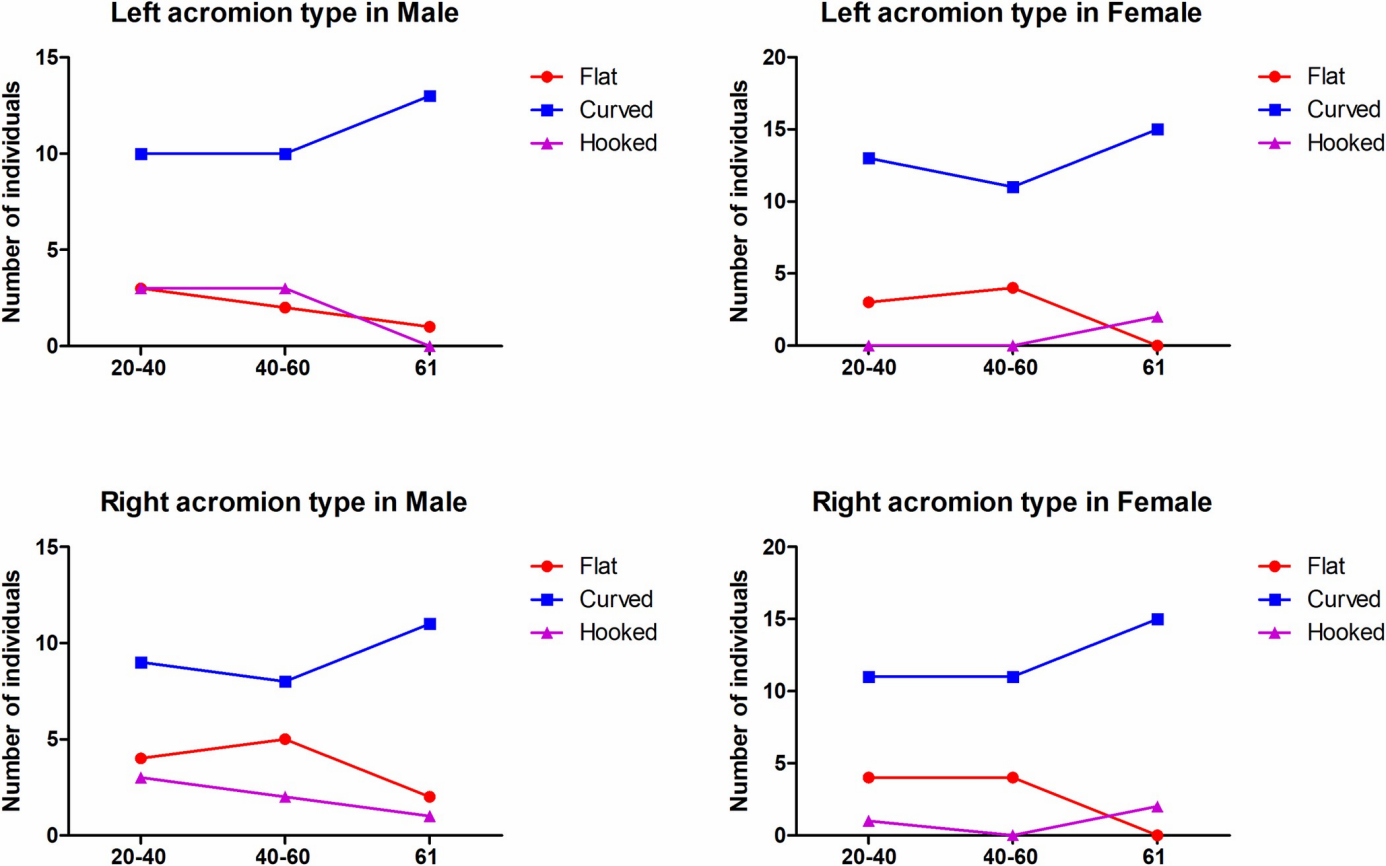
The acromial type in different sex and age groups. There were no significant differences in the acromial classification among the three different age groups.

The mean k value for the inter-observer reliability was 0.837, which indicated satisfactory agreement. The type of acromion in the three different age groups is shown in **Fig 5**. Type II (curved) was the most common type of acromion in the present study, accounting for 77.42% of left shoulders and 69.89% of right shoulders, and the hooked shape was a rare form, accounting for only 8.6% and 9.68% of left and right shoulders, respectively. There were no significant differences in the acromial classification among the three different age groups.

### Spur distribution and morphology

**Fig 6:**
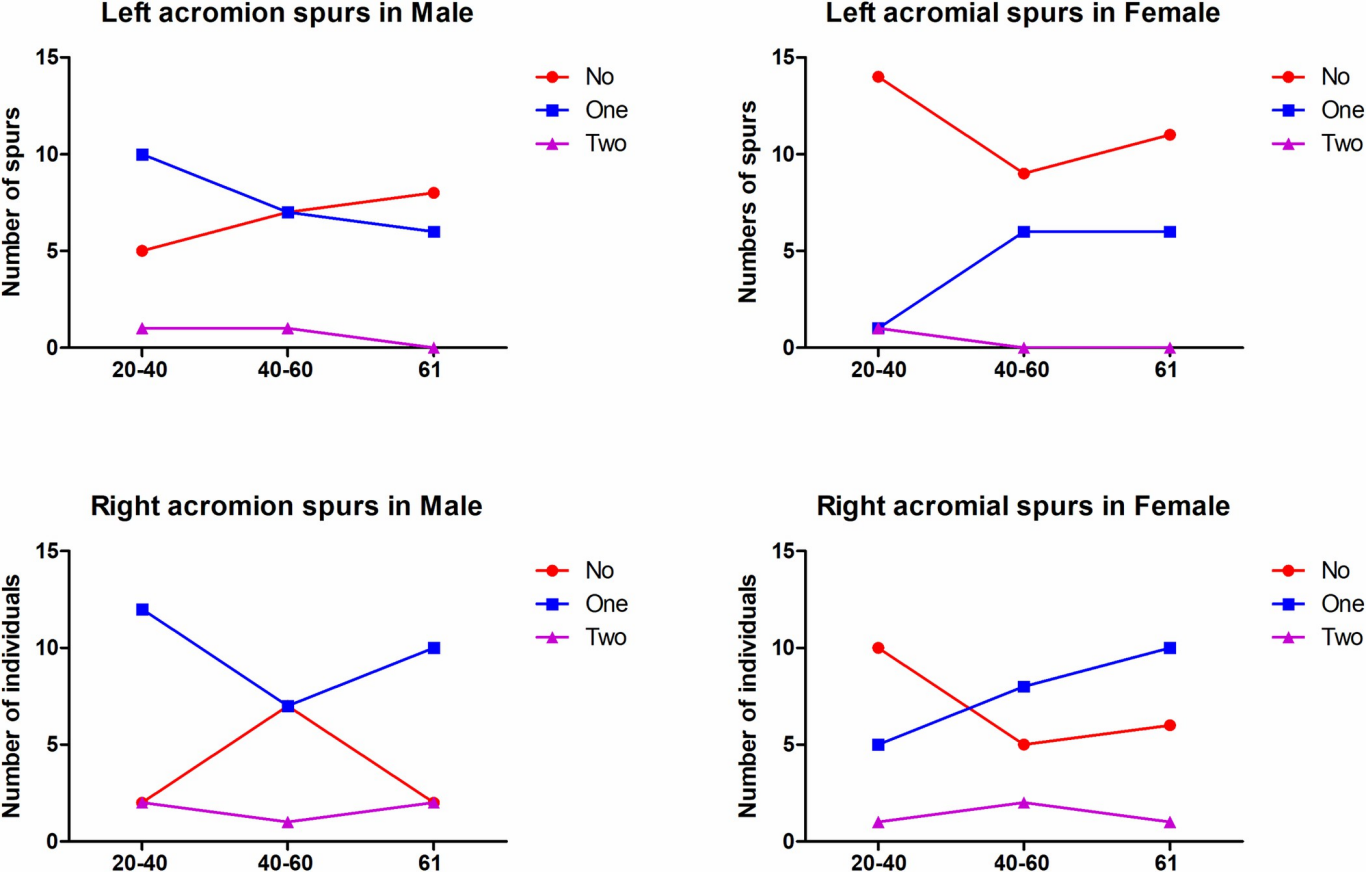
The number of acromial spurs in different sex and age groups. Males in group I were more prone to have subacromial spurs than females in the left and right shoulders (*P* = 0.003, P = 0.014), and a significant increase in the occurrence of spurs was also found in males compared with females in the right shoulder (*P* = 0.014).

The distribution of the number of spurs in the three different age groups is shown in **Fig 6**. Among the 93 patients evaluated, 54 (58.06%) were identified as having no spurs in the left shoulder, and the other 39 individuals (41.94%) were identified having spurs (36, one spur; 3, two spurs) in the left shoulder. Regarding the right shoulder, 32 (34.41%), 52 (55.91%) and 9 (9.68%) patients were identified as having no spurs, one spur and two spurs, respectively. Significantly fewer spurs were observed in the left shoulder than in the dominant right shoulder (*P* = 0.0482).

There were no significant differences in the number of subacromial spurs in males or females among the three different age groups. However, males in group I were more prone to have subacromial spurs in the right should than females (*P* = 0.003), and a significant increase in the occurrence of spurs in the right shoulder was also found in males compared with females in group I (*P* = 0.014). The distribution of spurs varied by acromial type. The existence of spurs was increased among the nonflat type of acromion on the dominant (right) side (X^2^ = 4.375, *P* = 0.036 <0.05). Spurs distributed in the A+B region were more commonly observed in young males and older females, but no differences were illustrated in the subacromial spur location among the different age groups (Table 1).

**Table 1:** The distribution character of all subacromial spur.

	Left shoulder (N)			Right shoulder (N)		
**Male**	A	B	A+B	A	B	A+B
20–40	1	3	8	1	3	13
41–60	0	1	8	3	1	5
>61	1	0	5	1	0	5
**Female**						
20–40	1	0	2	0	3	4
41–60	0	0	6	2	1	9
>61	1	3	13	4	0	8

There was a significant increase in the male subacromial surface area in the left shoulder in group III compared with group I (*P* < 0.001) and group II (*P* = 0.004). The total ratio of the spur/subacromial area was significantly higher in group II than in group I. For the right shoulder, an obvious increase in the subacromial area was observed in group III compared with group I (*P* = 0.004). Furthermore, there was a significant increase in the female spur area (*P* = 0.021) and the total ratio of the spur/subacromial area (*P* = 0.006) in the right shoulder in group II compared with group I (Table 2).

**Table 2:** The area of subacromial surface and total spur area.

	Left shoulder area (mm^2^)			Right shoulder area (mm^2^)		
**Male**	Subacromial	Main spur	Ratio	Subacromial	Main spur	Ratio
20–40	1199.88±141.46	158.55±72.48	0.14±0.05	1280.44±154.28	172.73±73.44	0.15±0.06
41–60	1094.94±93.80	199.62±80.11	0.19±0.06^a^	1361.76±186.25	198.78±112.32	0.15±0.07
>61	1423.51±179.95^a,b^	190.70±47.49	0.14±0.05	1492.58±185.09^a^	194.30±76.15	0.14±0.07
**Female**						
20–40	1017.50±43.79	107.18±3.69	0.12±0.01	991.00±147.61	109.93±52.68	0.12±0.06
41–60	880.58±187.38	136.12±82.31	0.17±0.11	947.22±191.18	187.14±56.25 ^a^	0.23±0.06 ^a^
>61	891.26±126.00	156.60±85.89	0.18±0.09	894.87±103.72	159.84±67.70	0.18±0.08

Note: a, compared with group 1(20–40) b, compared with group 2 (41–60).

### Acromial angle and acromial classification

**Fig 7:**
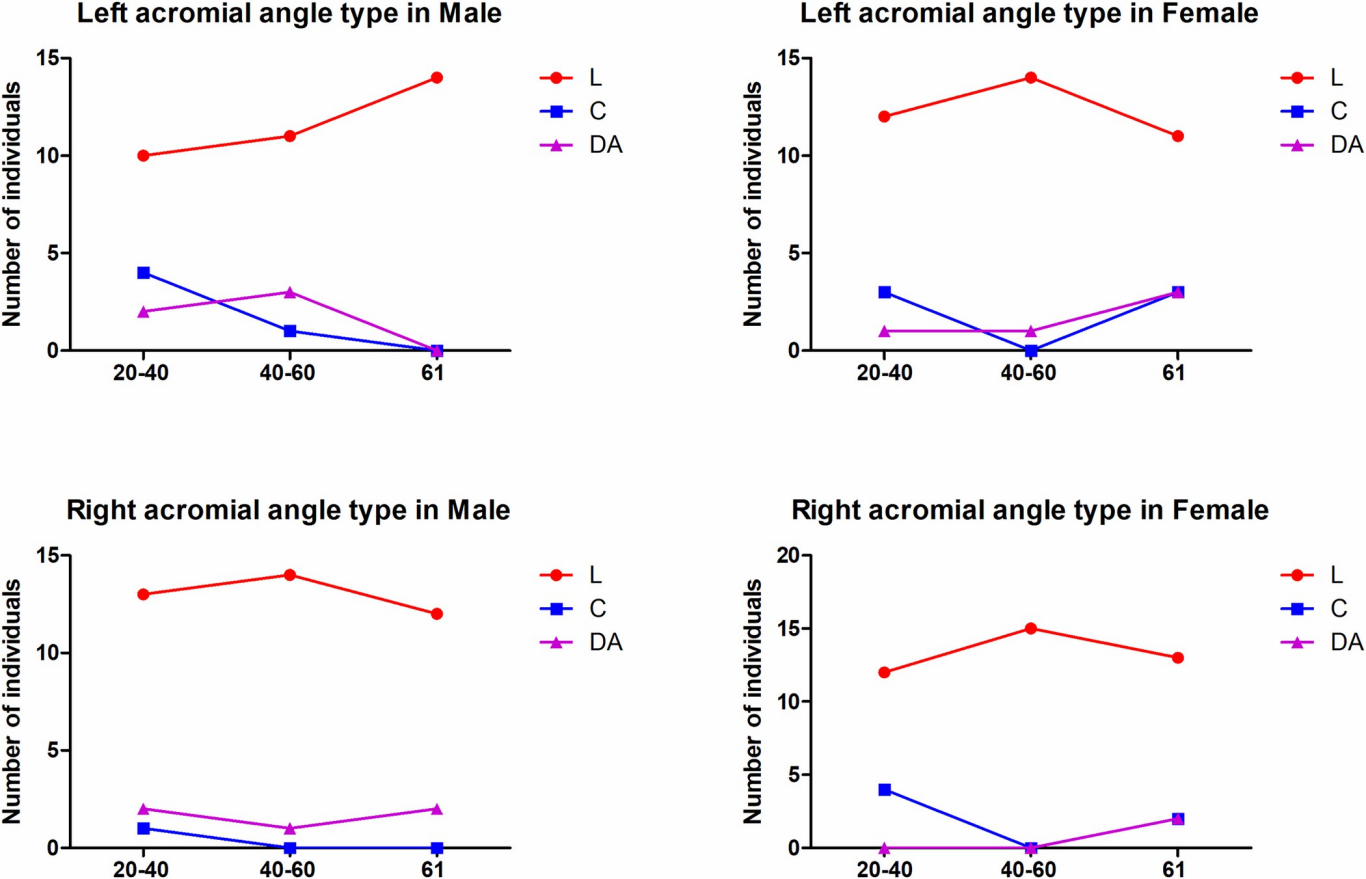
The acromial angle type in different sex and age groups. There were no significant differences in the acromial angle in males compared with females among the three different age groups. More L- or double angle-shaped acromial angles were found in the left shoulder in females than in males in group III (≥61 years).

The type of acromial angle in the three different age groups is shown in **Fig 7**. The L shape was the most common acromial pattern and accounted for 77.42% and 84.95% of the left and right shoulders, respectively, whereas the C (11.83% in left, 7.53% in right) and double angle (10.75% in left, 7.53% in right) shapes were rare. There were no significant differences in the acromial angle in males compared with females among the three different age groups. More L-shaped or double-angle acromial patterns were found in the left shoulder in females than males in group III (≥61 years), but no significant differences in the acromial angle classification were illustrated in males compared with females in the right shoulder or in the left shoulder in other age groups.

The double angle-shape pattern was the most common among type II acromions (curved shape, 8/10, 80%) on the left side. A curved acromion with an L-shaped acromial angle was the most commonly observed type in males and females. An L-shaped pattern was most common among type II acromions (curved shape, 56/78, 71.8%) on the right side. A curved acromion with an L-shaped acromial angle was the most commonly observed type in males and females. No significant differences were found between the different acromial angle patterns or acromial types (Table 3).

**Table 3:** The association between the acromion classification and acromial angle.

	Left shoulder			Right shoulder		
**Male**	Flat (%)	Curved (%)	Hooked (%)	Flat (%)	Curved (%)	Hooked (%)
L shape	5	27	3	8	25	6
C shape	0	3	2	0	1	0
DA	1	3	1	2	3	0
**Female**						
L shape	6	29	2	7	31	1
C shape	1	5	0	1	4	2
DA	0	5	0	0	2	0
**Total**						
L shape	11 (15.28)	56 (77.78)	5 (6.94)	15 (19.23)	56 (71.79)	7 (8.94)
C shape	1 (9.09)	8 (72.73)	2 (18.18)	1 (12.5)	5 (62.5)	2 (25)
DA	1 (10)	8 (80)	1 (10)	2 (28.57)	5 (71.43)	0

### Subacromial spur and acromial morphology classification

One spur was most common in type II acromions (curved shape, 29/36) (80.56%) on the left side; a curved acromion without spurs was the most commonly observed type in females, and a curved acromion with one spur was the most commonly observed type in males. On the right side, one spur was most common in type II acromions (curved shape, 34/52, 65.38%); a curved acromion with one spur was the most commonly observed type in males and females (Table 4).

**Table 4:** The association between the acromion classification and subacromial spurs.

	Left shoulder			Right shoulder		
**Male**	Flat (%)	Curved (%)	Hooked (%)	Flat (%)	Curved (%)	Hooked (%)
No Spurs	5	14	1	3	7	1
One Spurs	1	17	5	8	16	5
Two Spurs	0	2	0	0	5	0
**Female**						
No Spurs	6	26	2	3	17	1
One Spurs	1	12	0	3	18	2
Two Spurs	0	1	0	2	2	0
**Total**						
No Spurs	11 (20.37)	40 (74.07)	3 (5.56)	6 (18.75)	24 (75)	2 (6.25)
One Spurs	2 (5.56)	29 (80.56)	5 (13.89)	11 (21.15)	34 (65.38)	7 (13.46)
Two Spurs	0	3	0	2 (22.22)	7 (77.78)	0 (0)

## Discussion

When subacromial compression is suspected, understanding the morphology of the undersurface of the acromion is essential. Neer described the presence of a proliferative “spur” or ridge on the undersurface of the acromion (anterolaterally), which was recognized as the cause of “impingement”, as it reduced the subacromial space and therefore made it easier for the RC to come into contact the acromion. The subacromial space is traditionally defined by the humeral head inferiorly, the anterior edge and undersurface of the anterior third of the acromion, coracoacromial ligament, and the acromioclavicular joint superiorly [12]. After observing the morphology of osteophytes in our research, it was noticed that the osteophytes were not just distributed on the anterolateral side of the acromion; many subjects had subacromial osteophytes that were always located in or involved other areas of the acromion (posterior or anterior medial). Therefore, acromion decompression should not be limited to the anterior part of the acromion and should be enlarged to include or focused on other parts of the subacromial surface in some patients. As such, subacromial decompression should be based on the needs of individual patients.

A greater understanding of subacromial spurs is not only beneficial to assist the detection of SIS which was confirmed by radiological investigation, but can also guide the localization of lesions during arthroscopic surgery. The location of RC tears is often closely related to the type and location of subacromial spurs. Several studies have identified that a heel-like spur is a risk factor for bursal-side partial-thickness RC tears and full-thickness RC tears, while traction-like spurs greater than 5 mm are also considered a risk factor for full-thickness RC injuries [24–26]. The most common site of RC tears is the supraspinatus tendon, but the infraspinatus and the long head of the biceps brachii are also common sites of tears; thus, the site of tears, especially intratendinous tears, needs to be identified by experienced surgeons. It is usually possible to quickly locate lesions based on the specific location or morphology of acromion spurs during the decompression procedure, which is key to RC repair. Therefore, it is beneficial to perform RC repair after conducting acromioplasty. Furthermore, Joo [4] proposed the acromion process cross-sectional area as a sensitive parameter for assessing SIS, but it is believed that the subacromial surface flatness and morphological character of spurs are also important indicators for predicting the occurrence of SIS. It was found that the total spur/subacromial area ratio in the left and right shoulders was greater in group II than in group I, which is due to the increased age, more frequent shoulder joint activity or physical labor and greater bone hyperplasia of patients in group II. However, there was no significant difference in the ratio between group III and group I or II. The reason for this finding may be that the rate of bone spurs below the acromion is reduced with the occurrence of osteoporosis. While it is considered that increased age is one of the main reasons for the development of SIS and that the probability of SIS caused by spurs decreases with further advancing age, this does not mean that the probability of RC injury is low. In contrast, it is very likely that the risk of RC tears will gradually increase with age, and further study is needed to explore other factors affecting RC injury. Therefore, this study helps to deepen surgeons’ understanding of the distribution of acromial spurs; that is, parts of the subacromial acromioplasty procedure should be carried out in each individual subjects according to individualized differences.

Studying the surface flatness or the distribution of acromial spurs is helpful to clarify the relationship between acromial spurs and acromial classification types. The acromion is traditionally classified into three types; among them, the flat and curved types are the most commonly observed, while the hook type is relatively less common. Additionally, it is often considered that large bony spurs in the anterolateral acromion exist. The results of this study suggest that the right shoulder is more prone to have spurs than the left shoulder and that acromions with spurs are more prone to be of the curved type. Furthermore, the A+B type distribution of bone spurs was more frequently observed, and while B type spurs were mainly distributed anteriorly, they were not limited to the anterolateral region but were also likely located in the middle or even the anteromedial region (A type) of the acromion. Studies have confirmed that the anterolateral prominence is a native normal structure that naturally forms corresponding to the traction of the coracoacromial ligament. Similarly, some studies have proven that the anterolateral prominence can be observed in all asymptomatic and cadaveric patients. Furthermore, some studies have denied the necessity of decompression in patients diagnosed with SIS, but full decompression is still needed for anterior or posterior spurs based on our clinical experience and follow-up. Studies have shown that the curved or hooked portion of the acromion is not always visualized on the supraspinatus outlet view. This view is more accurate for the discovery of spurs in the middle and lateral regions, but its accuracy is not high for medial spurs [27]. In our study, by collecting CT data and using Mimics software for 3D reconstruction of the shoulder, the shape of spurs under the acromion could be observed more intuitively. Observation of the 3D images indicated that the presence of a hooked acromion does not limit the potential location of spurs to only the anterolateral region but that spurs may potentially exist in the middle or even medial region.

An uneven acromial surface is so common in normal people that acromioplasty should be performed in the majority of RC injury cases accompanied by SIS. In addition, the relationship between the acromial angle and acromial classification is not definite, but research on the acromial angle is helpful to accurately plan the posterior surgical approach. The measurement of spur characteristics, including location and surface area, is helpful for conducting acromioplasty. The results of this study showed that the right shoulder spur/subacromial surface area of females was significantly increased in group II compared with group I. Other studies have also reported that people in their 40s-60s are more likely to have RC injuries than younger people [28]. The time required for acromioplasty using a burr with a diameter of 4 mm and a working area approximately 0.5 mm^2^ was evaluated. According to the efficiency of the burr (3 s for each burr) manipulated by an experienced orthopedic surgeon, the time required for subacromial acromioplasty should be approximately 19.8 mins (198.78 mm^2^ in group II). For patients with SIS, because of the potential for larger spurs, the time required for acromioplasty may be longer; a complete procedure should consider all possible factors of impingement, the range of removal should not be limited to the anteroinferior surface of the acromion, and spurs in the supraspinatus and infraspinatus channels should be removed as much as possible in our opinion. The conventional anterolateral working approach, with observation through the posterior approach, can be used to completely remove spurs from the anterior and posterior surface of the subacromial peak.

One limitation of this research is that the enrolled subjects were normal and asymptomatic; thus, the results cannot indicate the course of spurs in patients with SIS. However, SIS can be observed in both asymptomatic and symptomatic people; therefore, this work still offers certain guiding significance for the morphological study of subacromial spurs in patients with SIS. Second, SIS can not only be caused by contact between the acromion and RC but can also originate from the RC tendons or be mediated by the free nerve endings in the bursa [12,29]. In a follow-up study, the mechanism and scope of action of bone spurs and the RC itself in shoulder pain or SIS will be further clarified. Third, the inclusion of patients with RC injury along with impingement in future research would be more helpful to clarify the relationship between the shape of the spur and the location of the RC injury. Regarding the effect of individualized acromioplasty, long-term follow-up will be conducted to clarify the efficacy in patients with different forms of spurs.

## Conclusion

Subacromial spurs are mainly distributed with an irregular shape and mostly run through the medial and lateral regions of the subacromial surface in normal subjects. The characteristics of subacromial spurs are so diverse that the surgeon must conduct subacromial decompression completely based on the individual morphological characteristics of spurs.

## Supporting information

S1 Video The subacromial surface area was defined and isolated with 3 Matic. (MP4)

S2 Video The relevant parameter about the subacromial surface area was calculated with 3 Matic. (MP4)

S1 Data (XLSX)

## Abbreviations

SIS: Shoulder Impingement Syndrome
RC: Rotator Cuff
